# KIF14 affects cell cycle arrest and cell viability in cervical cancer by regulating the p27^Kip1^ pathway

**DOI:** 10.1186/s12957-022-02585-3

**Published:** 2022-04-19

**Authors:** Jie Zhang, Gulimire Buranjiang, Zuohelaguli Mutalifu, Hua Jin, Liyan Yao

**Affiliations:** 1 Department of Obstetrics and Gynecology, Second Affiliated Hospital of Xinjiang Medical University, Nanhu Road, Urumqi, Xinjiang 830011 China; 2 Department of Obstetrics and Gynecology, Second Affiliated Hospital of Xinjiang Medical University, Nanhu Road, Urumqi, Xinjiang 830063 China

**Keywords:** Cervical cancer, KIF14, Cell cycle arrest, p27, Degradation

## Abstract

**Background:**

Cervical cancer is a kind of malignant gynecological tumor. The first choice for treating cervical cancer is still a combination of surgery and chemoradiotherapy, but the 5-year survival rate remains poor. Therefore, researchers are trying to find new ways to diagnose and treat cervical cancer early.

**Methods:**

The expression level of KIF14 in cells and tissues was determined via qRT–PCR. The ability of the cells to proliferate, migrate, and invade was examined using CCK-8 assay kits, colony formation assays, and Transwell chambers. The expression levels of Cyclin D1, Cyclin B1, p21, and p27 were also detected using western blot assays.

**Results:**

The results suggested that p27 is a key regulatory factor in the KIF14-mediated regulation of the cell cycle. In addition, KIF14 knockdown promotes malignancy in cervical cancer cells by inhibiting p27 degradation, resulting in cell cycle arrest.

**Conclusions:**

KIF14 is an oncogene in cervical cancer, and knocking down KIF14 causes cell cycle arrest by inhibiting p27 degradation, thus affecting cell viability, proliferation, and migration. These results provide a potential therapeutic target for cervical cancer.

## Background

Cervical cancer is one of the most common malignant gynecological tumors: it accounts for approximately 270,000 deaths every year, ranking fourth in cancer mortality among females globally [1, 2]. It has become a prominent public health problem and seriously threatens the health of women worldwide [3]. Advances in screening technology and the use of human papillomavirus (HPV) vaccines in clinical practice have resulted in great progress in the prevention and treatment of cervical cancer. However, due to the cost and technical requirements of screening, these tests cannot be widely used in developing countries. In addition, although the combination of surgery and chemoradiotherapy is still the first choice for cervical cancer treatment, the 5-year survival rate after treatment is less than 40% [4, 5]. It is still vital to study the pathogenesis of cervical cancer and find markers that predict specific therapeutic targets.

Kinesin is a molecular motor that performs its biological function by moving along microtubules [6]. It modulates the transport of biological macromolecules to ensure the basic activity of cells, plays a role in the regulation of some intracellular molecular signaling pathways, and extensively participates in many diseases [7, 8]. KIF14 belongs to the kinesin superfamily and participates in chromosome separation and cytokinesis [9, 10]. Studies have found that KIF14 is associated with the occurrence of various cancers [11–13]. Therefore, this study aimed to analyze the possible relationship between KIF14 and cervical cancer.

The cell cycle is the center of cellular activity, and the abnormal regulation of the cell cycle is closely associated with tumor development and malignancy [14], the inactivation of suppressor oncogenes, and the alteration of apoptosis-regulating genes or mutated genes [15, 16]. Regardless of the cause, the ultimate manifestation of various cancers is the abnormal growth of normal cells. Cell cycle regulation is the most common molecular mechanism underlying tumor formation [17]. p27^Kip1^ (hereafter p27) is a member of the cyclin-dependent kinase inhibitor (CDKI) family. It suppresses cyclin/CDK complex activity and arrests the cell cycle [18]. Moreover, decreases in p27 are positively correlated with the rate of apoptosis [19].

Here, we verified the role and regulatory mechanism of KIF14 in cervical cancer. Collectively, our findings reveal the functional mechanism by which KIF14 regulates the cell cycle during tumorigenesis, thus providing potential value in the treatment of cervical cancer.

## Materials and methods

### Cell culture

The human cervical cancer cell lines HeLa, Ca Ski, and SiHa and the cervical epithelial cell lines H8 and HUCEC (Institute of Cell Biology, China) were cultured in RPMI-1640 medium (HyClone, USA) supplemented with 10% FBS (Gibco, USA). All the cell lines were maintained in a humidified incubator containing 5% CO_2_ at 37 °C.

### Tissue sample collection

Human cervical tissue samples and normal cervical tissue samples were collected by professionals and preserved in the specimen bank of the Cancer Prevention and Treatment Institute of the Affiliated Cancer Hospital of Xinjiang Medical University.

### Cell transfection

Cells were seeded into 6-well plates and cultured as described above. The culture medium was replaced after 6 h. For transient silencing, cells were transfected with KIF14 siRNA or control siRNA duplexes (Sangon Bioengineering, China). The details of the interference sequences are as follows: siKIF14-1: 5′-GCCCGUUUAAUAGUCAACAUUTT-3′; siKIF14-2: 5′- GCUGCAUUUGAAGUCGGAUAUTT-3′; siKIF14-3: 5′-CGGCAAGAAAUAACAUCCUUATT-3′; and siNC: 5′-UUCUCCGAACGUGUCACGUTT-3′. Cells were transfected with Lipofectamine 8000 (Beyotime, China) according to the manufacturer’s instructions.

### qRT–PCR

Tissue and cell samples were lysed with TRIzol (Invitrogen, USA). A TaqMan Reverse Transcription kit (Takara, Japan) was used to reverse transcribe cDNA. Reactions were run on an ABI 7000 Thermocycler (Applied Biosystems, USA). The expression of each gene was calculated using the 2^−ΔΔCt^ method. The following primer sequences were used: KIF14 forward, 5′-CGGAACAAGCAAACCAAAGGAGTG-3′, reverse, 5′-GCAGCGGGACTAATCGTAGCAATC-3′; Cyclin D1 forward, 5′-CAGAGGCGGAGGAGAACAAA-3′, reverse, 5′-ATGGAGGGCGGATTGGAA-3′; Cyclin B1 forward, 5′-ATGCAGCACCTGGCTAAGAA-3′, reverse, 5′-TACACCTTTGCCACAGCCTT-3′; and GAPDH forward, 5′- GGGTGATGCAGGTGCTACTT-3′; reverse, 5′-GCACTTGGGGCAGAGATGAT-3′.

### Western blot

Cells were collected and lysed with RIPA buffer (Beyotime, China). Lysates (20–30 μg) were added to SDS–PAGE gels and transferred to PVDF membranes. Blots were probed with primary antibodies (Cell Signaling Technology, USA) against KIF14, Cyclin D1, Cyclin B1, p21, or p27, overnight at 4 °C. Membranes were then incubated with the corresponding HRP-conjugated secondary antibodies for 1 h. The blots were subsequently detected with an ECL Kit (Beyotime, China). Protein blotting images were analyzed using ImageJ software (NIH, USA).

### Cell viability

The cells in each transfected group were digested with a routine trypsin protocol and added to serum-free medium that had been precooled at 4 °C. After centrifugation, a 100-μl cell suspension was prepared in a 96-well plate. At 24 h, 48 h, and 72 h, 10 μl CCK8 solution (Dojindo, Japan) was added to each well. The absorbance at 450 nm was measured with a microplate analyzer (Biotek Instruments, USA).

### Cell migration and invasion assays

To assess cell migration and invasion, Transwell assays were performed as previously described [20]. Forty-eight hours after transfection, 5 × 10^4^ cells were resuspended in serum-free media and then inoculated in the upper chamber. For the invasion assay, Matrigel (BD Biosciences, USA) was added to the upper chamber. Medium with 10% FBS was added to the lower chambers. After 24 h, the cells were fixed and stained with 0.5% crystal violet (Solarbio, China) for 20 min. A microscope (Leica Microsystems, Canada) was used to capture images.

### Statistical analysis

All the results were analyzed using the SPSS 21.0 software (IBM, USA). The data are from at least three independent repeats and are shown as the mean ± SD. Statistical tests were performed using Dunnett’s *t* test and one-way analysis of variance (ANOVA) for more than two groups. *P* < 0.05 was considered statistically significant.

## Results

### KIF14 is highly expressed in cervical cancer

**Fig. 1:**
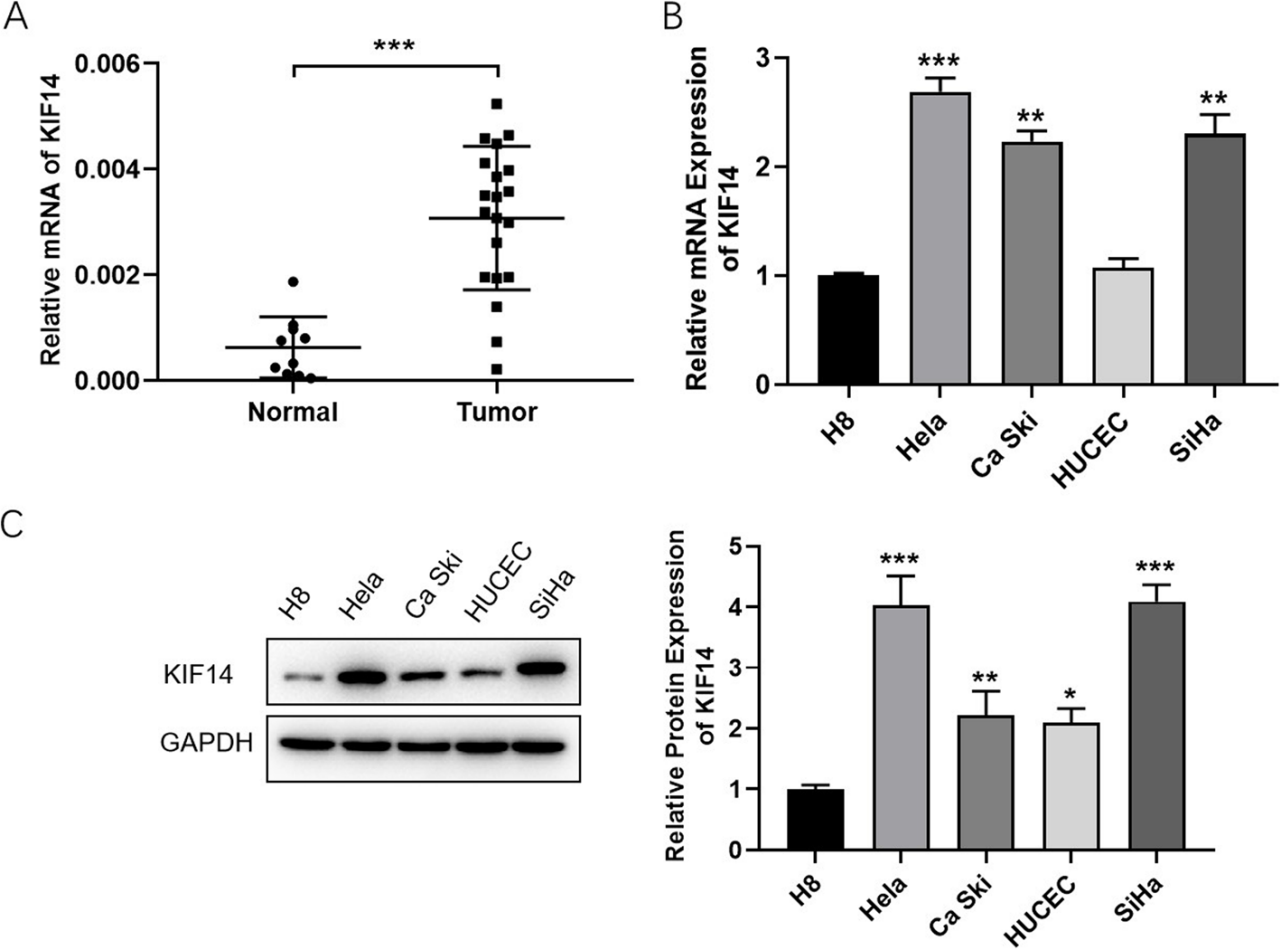
KIF14 is highly expressed in cervical cancer. **A** KIF14 levels in cervical cancer tissues were detected via qRT–PCR. **B**, **C** The relative mRNA and protein levels of KIF14 in cervical cancer cells; cervical cells were assessed via RT–qPCR and western blot assays. *p < 0.05, **p < 0.01, and ***p < 0.001

RT–qPCR showed that the KIF14 level was upregulated in 20 cervical cancer tissues compared to 10 normal tissues (Fig. 1A). In addition, the KIF14 level in cervical cancer cells was higher than that in cervical cells (Fig. 1B, C). Therefore, we concluded that KIF14 was upregulated in cervical cancer.

### KIF14 knockdown inhibits cell proliferation, migration and invasion

**Fig. 2:**
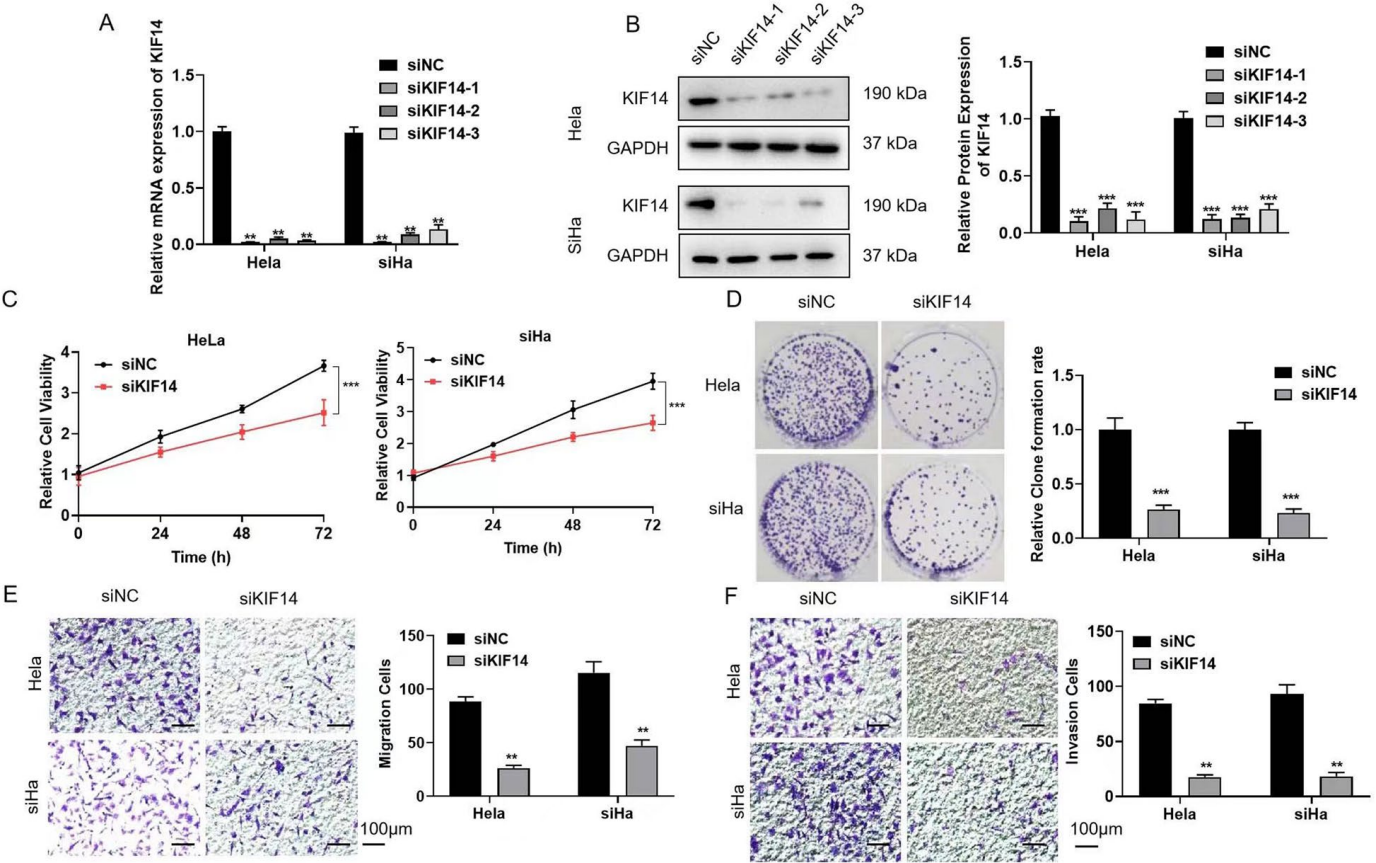
KIF14 knockdown inhibits cell proliferation, migration and invasion. HeLa and siHa cells were transfected with siNC, siKIF14-1, siKIF14-2, or siKIF14-3 for 24 h. **A**, **B** Relative mRNA and protein levels of KIF14 in cervical cancer cells from the interference groups. **C** CCK-8 assays detected the cell viability in the KIF14 interference groups. **D** A clone formation assay was used to detect the proliferative ability. **E** Detection of cell migration ability. Scale bar = 100 μm. **F** Detection of cell invasion ability. Scale bar = 100 μm. *p < 0.05, **p < 0.01, and ***p < 0.001

To study the effects of KIF14 in cervical cancer, three pairs of siRNA sequences, siKIF14-1, siKIF14-2, and siKIF14-3, were designed against the target KIF14 gene sequence, and siNC was designed for the control transfection group. Then, we verified the transfection efficiency and detected the levels of KIF14 mRNA and protein after transiently transfecting HeLa and siHa cells with the interference sequences (Fig. 2A, B). A CCK-8 assay showed that the cell viability in the interference group was significantly inhibited (Fig. 2C). The results of a clone formation experiment are shown in Fig. 2D. Transwell experiments were used to detect cell migration and passage through Matrigel in the control and interference groups. Interfering with KIF14 expression significantly inhibited the migration and invasion of cervical cancer cells (Fig. 2E, F).

### KIF14 overexpression promotes cell proliferation, migration, and invasion

**Fig. 3:**
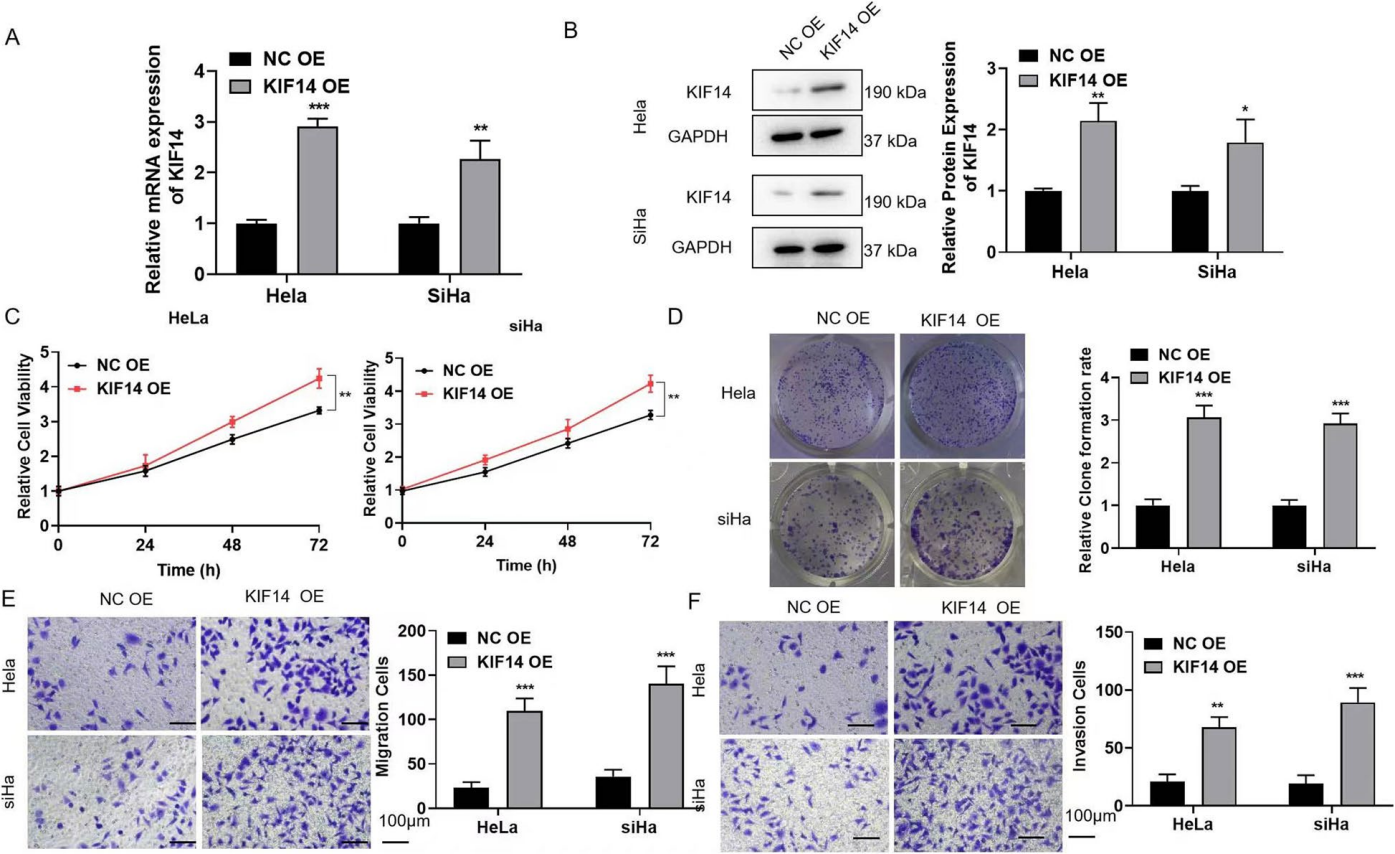
KIF14 overexpression promotes cell proliferation, migration, and invasion. HeLa and siHa cells were transfected with NC OE or KIF14-3 OE for 24 h. **A**, **B** The transfection efficiency of KIF14 OE C. Cell viability was detected via a CCK-8 assay. **D** A clone formation assay detected the proliferation ability. **E** Detection of cell migration ability. Scale bar = 100 μm. **F** Detection of cell invasion ability. Scale bar = 100 μm. *p < 0.05, **p < 0.01, and ***p < 0.001

The effect of KIF14 overexpression on cervical cancer cells was further studied, and we first verified the overexpression efficiency in HeLa and siHa cells. The relative mRNA expression of KIF14 was higher in the KIF14 OE group than that in the NC group (Fig. 3A). In addition, the trends in protein and mRNA expression were the same (Fig. 3B). A CCK-8 assay showed that cell proliferation was significantly increased in the KIF14 OE group (Fig. 3C). Clone formation and Transwell assays suggested that KIF14 overexpression could significantly promote the proliferation, migration, and invasion of cervical cancer cells (Fig. 3D–F).

### KIF14 knockdown results in cervical cancer cell cycle arrest

**Fig. 4:**
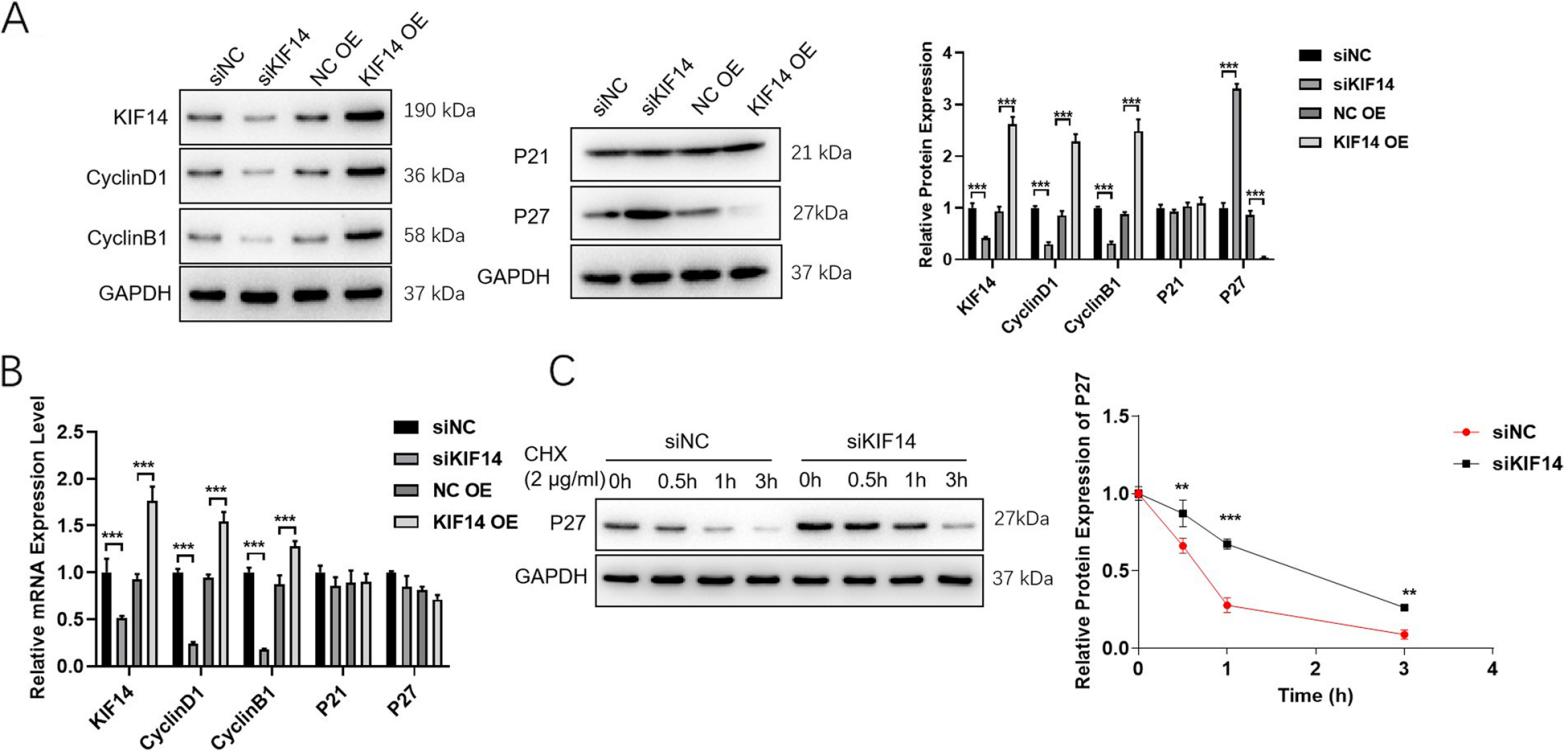
KIF14 knockdown results in cervical cancer cell cycle arrest. HeLa cells were transfected with si-NC, si-KIF14, NC OE, or KIF14 OE for 24 h. **A** The KIF14 and cyclin-associated protein levels were detected via western blot. **B** KIF14 knockdown or overexpression and the mRNA levels of Cyclin D1, Cyclin B1, p21, and p27. **C** HeLa cells were transfected with si-NC or si-KIF14 for 24 h and then treated with 2 μg/ml cycloheximide for 0 h, 0.5 h, 1 h, and 3 h. KIF14 knockdown inhibited p27 degradation. *p < 0.05, **p < 0.01, and ***p < 0.001

There is a close relationship between the cell cycle and tumor progression. When the regulatory mechanism of the cell cycle is damaged, normal cell growth can be uncontrolled; thus, cells can transform into tumor cells. Cells were divided into siNC, siKIF14, NC OE, and KIF14 OE groups. As shown in Fig. 4A, KIF14 inhibition suppressed KIF14, Cyclin D1, and Cyclin B1 levels and increased p27 levels. While overexpressing KIF14 in HeLa cells increased the levels of KIF14, Cyclin D1, and Cyclin B1, it reduced the p27 protein level. Interestingly, neither the knockdown nor the overexpression of KIF14 affected p27 mRNA expression levels, which was inconsistent with the changes in protein levels (Fig. 4B). These data indicated that p27 might be regulated at the level of protein stability. Thus, 2 μg/ml cycloheximide was added to assess p27 stability. As shown in Fig. 4C, KIF14 knockdown inhibited p27 degradation.

### KIF14 knockdown arrests the cell cycle by inhibiting p27 degradation

**Fig. 5:**
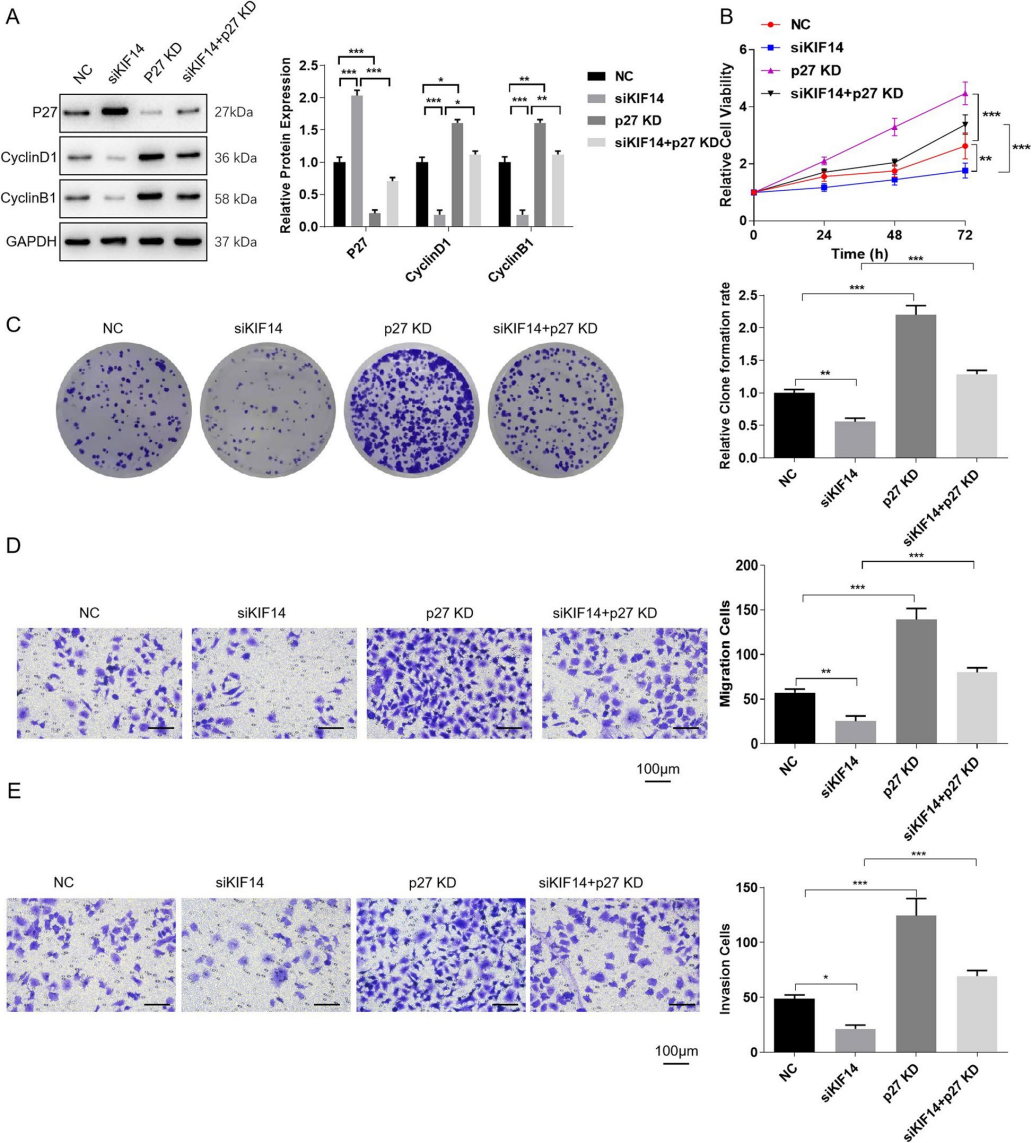
KIF14 knockdown arrests the cell cycle by inhibiting p27 degradation. HeLa cells were divided into siNC, siKIF14, p27 KD, and siKIF14+p27 KD groups. **A** The trends in the changes associated with KIF14 and cyclin-associated protein expression levels were reversed in the si-KIF14+p27 KD group compared with the si-KIF14 group. **B** Cell viability as detected via CCK-8 assays. **C** A clone formation assay was used to detect the proliferation ability. **D** Detection of cell migration ability. Scale bar = 100 μm. E. Detection of cell invasion ability. Scale bar = 100 μm. *p < 0.05, **p < 0.01, and ***p < 0.001

To further verify the regulatory mechanism by which KIF14 affects the cell cycle by regulating p27 degradation, cells were transfected with siKIF14, p27 KD, or siKIF14+p27 KD. Western blot assays showed that p27 KD suppressed p27 expression and increased Cyclin D1 and Cyclin B1 levels (Fig. 5A). Cyclin D1 and Cyclin B1 levels were decreased and p27 levels were increased when the cells were transfected with siKIF14, but the trend was reversed when the cells were transfected with siKIF14 + p27 KD (Fig. 5A). Functionally, p27 KD promoted cell viability, clone formation, migration and invasion; siKIF14 suppressed cell viability, clone formation, migration and invasion; and siKIF14 + p27 KD reversed the effects of siKIF14 (Fig. 5B–E).

## Discussion

More than half a century of effort has enabled the prevention and treatment of cervical cancer [21]. However, cervical cancer is still an important public health problem, especially in developing countries and regions. Advanced cases and recurrent cases after treatment are not uncommon [22]. Strategies to improve patient prognosis and quality of life deserve further study. It is reported that abnormal expression of important genes and alterations of signaling pathways are associated with cancer pathogenesis [23, 24]. For instance, Qin F et al. revealed a novel angiogenesis subtype classification in squamous cell carcinoma [23]. Li S et al. screened six prognostic hub genes by weighted gene co-expression network analysis (WGCNA) combined with LASSO Cox-PH analysis, which provided a new strategy for targeted therapy of cervical cancer [24]. The study aimed to explore the molecular mechanism of cervical cancer and find new tumor markers and biological methods to treat tumors .

Increasing evidence demonstrated that gene therapy is a promising candidate in the treatment of cancers [25, 26]. For instance, Lu X et al. found that targeting IGF1 by miR-186-3p suppressed tumorigenesis of cervical cancer [25]. Besides, Zou H et al. clarified that circ_0018289 inhibition might be a promising point for the development of novel anti-tumor strategies against cervical cancer [26]. KIF14 has been proven by numerous researchers to be an important oncogene in many cancers [27–30]. It can be used as a marker for detecting paclitaxel resistance during the treatment of ovarian cancer [31]. Therefore, as KIF14 becomes an increasingly important target for cancer therapy and prognostic monitoring markers, it is crucial to understand the mechanisms regulating its gene [32]. Therefore, this study aimed to observe KIF14 expression in cervical cancer and further explore the influence of changes in its expression on the proliferation, invasion, migration, and cycle of cervical cancer cells.

In this study, the KIF14 level in cervical cancer tissue was significantly upregulated. These results suggested that KIF14 has great potential as a diagnostic and therapeutic target. Next, to explore the role of KIF14, we designed and synthesized three interference sequences targeting KIF14 and transiently transfected HeLa and SiHa cervical cancer cells. We observed transfection by using fluorescent groups coupled with the interference sequences and confirmed that the transfection efficiency met the requirements. The results showed that the three designed interference sequences could effectively interfere with the level of KIF14 at the gene level, but only SiKIF14-2 and SiKIF14-3 had significant interference efficiency at the protein level. When the expression of KIF14 was disturbed, cell proliferation, migration, and invasion were significantly inhibited. The results of these in vitro experiments all indicate that KIF14 is indeed a potential oncogene.

Some researchers have speculated that KIF14 is associated with cytokinesis and plays a key role in cell cycle arrest [33, 34]. Thus, we detected whether the cyclin-related proteins Cyclin D1, Cyclin B1, p21, and p27 were overexpressed or knocked down. After interference with the SiKIF14-2 sequence, the expression of Cyclin D1 and Cyclin B1 decreased, while the expression of p27 protein increased in HeLa cells; the trend in the change was the opposite in the case of overexpression. However, the specific molecules that interact with KIF14 in cells and the molecular mechanisms that inhibit cell growth need to be further studied and determined. We found that p27 mRNA levels did not change, which contrasted with the change in protein level. We speculated that p27 might be regulated at the level of protein stability. We compared the stability of p27 transfected with or without SiKIF14 using cycloheximide to inhibit protein synthesis. These results collectively clarified that KIF14 inhibition arrested the cell cycle by suppressing p27 degradation. Furthermore, the above conclusions were verified by rescue experiments.

## Conclusions

In conclusion, the KIF14 level is upregulated in cervical cancer, and KIF14 knockdown suppresses cell proliferation, migration, and invasion, resulting in cell cycle arrest by inhibiting p27 degradation.

## Data Availability

The data used to support the findings of this study are available from the corresponding author upon request.
